# Spinal cord neurenteric cyst: clinical and diagnostic findings and long term follow-up in two dogs

**DOI:** 10.1080/01652176.2018.1542515

**Published:** 2019-01-24

**Authors:** Teresa Gagliardo, Daniele Corlazzoli, Marco Rosati, Swan Specchi, Luciano Pisoni, Sara Del Magno, Simona Pappagalli, Greta Galli, Gualtiero Gandini

**Affiliations:** a Department of Veterinary Medical Sciences, University of Bologna, Ozzano dell’Emilia, Italy;; b Veterinary Diagnostic Center Palermovet, Palermo, Italy;; c Roma Sud Veterinary Clinic, Rome, Italy;; d Section of Clinical and Comparative Neuropathology, Centre for Clinical Veterinary Medicine, Ludwig-Maximilians-Universität München, Munich, Germany;; e Department of Diagnostic Imaging, Veterinary Institute of Novara, Granozzo con Monticello, Italy

**Keywords:** Dog, canine, neurenteric cyst, spinal cyst, intradural-extramedullary cyst, intradural-intramedullary cyst

## Case 1

A 4-year-old entire female 25 kg Golden Retriever was referred because of 1-month history of an insidious onset and progressive course of hind limb gait abnormalities. The general physical examination was unremarkable. Neurological examination showed mild proprioceptive ataxia of the hind limbs and marked monoparesis of the right hind limb. Postural reactions were reduced in the left hind limb and absent in the right. Segmental spinal reflexes were normal. The clinical findings were consistent with a thoracolumbar (T3-L3) neuroanatomical localization of the lesion.

The main differential diagnoses included degenerative (intervertebral disc disease), congenital (arachnoid diverticulum, epidermoid cyst), inflammatory/infective (meningomyelitis of unknown origin, bacterial, fungal, viral, and parasitic myelitis), and neoplastic (including nephroblastoma, lymphoma) etiologies. The complete blood cell count and serum biochemical analyses were within normal limits.

Magnetic resonance imaging (MRI) of the thoracolumbar vertebral column and spinal cord was performed using a 0.17 T Magnet (Scan-MR; Esaote, Genova, Italy). Sequences included T1-weighted (T1-W) and T2-weighted (T2-W) images in transverse and sagittal planes. In addition, T1-W images in sagittal, transverse, and dorsal planes were obtained after intravenous administration of paramagnetic contrast media gadoversetamide (Optimark, 0.5 mmol/ml, Mallinckrodt, Dublin, Ireland) at 0.1 mmol/kg bodyweight (BW).

**Figure 1.**
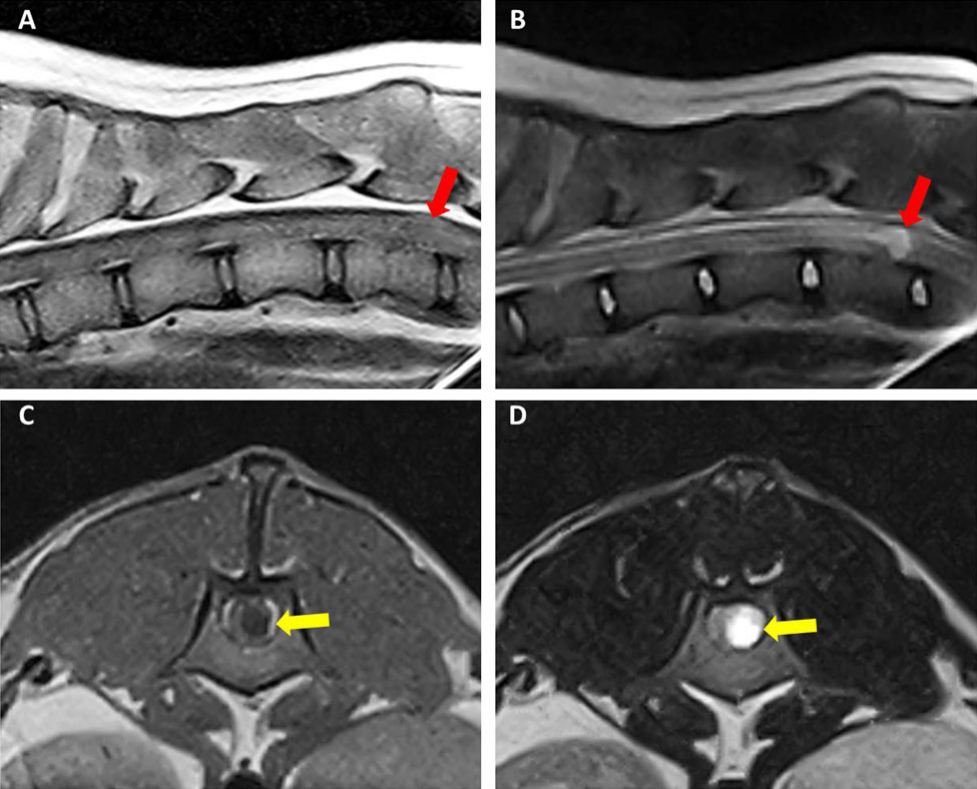
Case 1: sagittal T1-W (A) and T2-W (B) images of thoraco-lumbar spinal cord showing a roundish, well-demarcated lesion at the level of the 13th thoracic vertebra (red arrows). The lesion was iso- to slightly hypointense on T1-W images (A) and hyperintense on T2-W. Case 2: T1-W (C) and T2-W transverse (D) views of the apparently intradural-intramedullary cyst located at the level of L1 (yellow arrows). The content of the cyst appeared hyperintense in T2-W and hypointense on T1-W images.

MRI showed, at the level of the 13th thoracic vertebra, a lesion lateralized on the right side hyperintense on T2-W, iso- to slightly hypointense on T1-W images, with a mild peripheral contrast enhancement. The abnormal findings were consistent with a roundish, well-demarcated intradural-extramedullary spinal cord space-occupying lesion of 7 mm in diameter. MRI also showed mild dilatation of the central canal cranial to this mass consistent with mild syringohydromyelia, as illustrated in Figure 1.

Based on the MRI findings, the main differential diagnoses included an inflammatory space-occupying lesion, epidermoid cyst, and, less likely, juvenile neoplasia such as nephroblastoma or primary neuroectodermal tumor.

The dog underwent decompressive surgery under general anesthesia. Premedication was performed using acepromazine (0.02 mg/kg BW IM) and methadone (0.3 mg/kg BW IV), followed by induction with propofol (2 mg/kg BW IV). General anesthesia was maintained with isoflurane and oxygen. Intraoperative analgesia was provided by a constant rate infusion of fentanyl (0.005 mg/kg BW/h IV).

**Figure 2.**
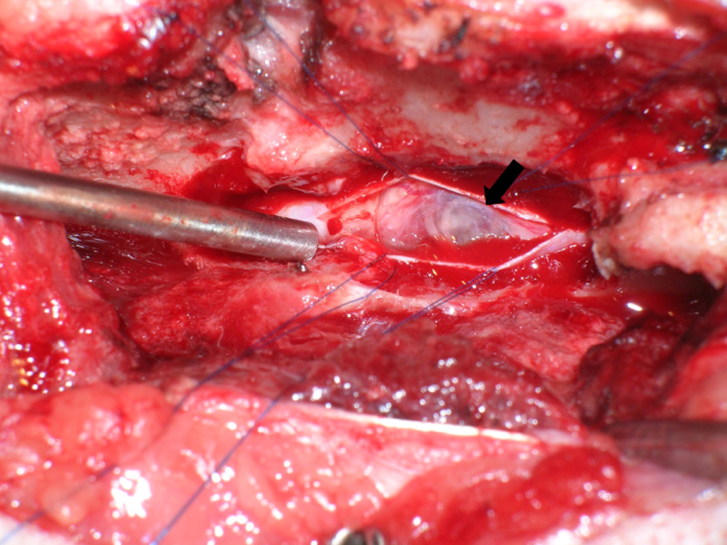
Intraoperative view of the spinal cord in case 1. After durotomy, a grayish white, fluid-filled mass was visualized (arrow).

A T13-L1 right hemilaminectomy was performed. Bulging of the dura mater was observed immediately following hemilaminectomy. After durotomy, the lesion appeared as a fluid-filled mass (Figure 2). The mass was not attached to the spinal cord, so it was removed *in toto* and submitted for histopathological examination.

All excised tissue was fixed in 10% neutral buffered formalin, embedded in paraffin, and sectioned. The sections were stained with hematoxylin and eosin, alcian blue, and periodic acid–Schiff (PAS).

**Figure 3.**
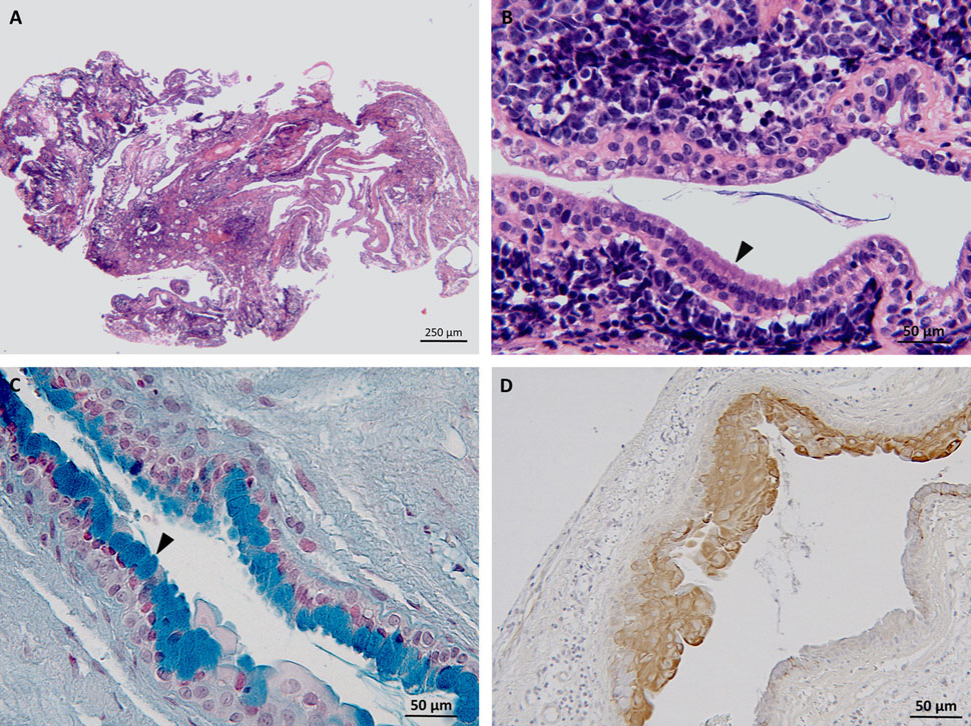
Case 1: (A) histology depicts a cystic and multiloculated, moderately cellular epithelial cell proliferation embedded in moderate amount of stroma, hematoxylin, and eosin stain (2.5×). (B) Higher magnification of (A) columnar epithelium lining an empty cavity and presenting with cilia (arrow head) consistent with respiratory epithelium, hematoxylin, and eosin stain (20×). Case 2: (C) Thin-layered epithelium within the neuroenteric cyst featuring multiple goblet cells containing acidic mucin (arrow head), Alcian blue stain (20×). (D) Immunohistochemistry for pancytokeratin showing a strongly immunoreactive pseudostratified to stratified cuboidal epithelium, DAB as chromogen (20×).

Histology showed non-encapsulated, partially cystic mass, consisting of moderately cellular epithelial proliferation. The cells were organized in ducts and occasionally branching tubules of cuboidal to columnar, mono-, or bilayered epithelium with intercalating goblet cells and occasional cilia on the luminal surface (Figure 3). The stroma presented some clefts and empty cavities lined by flattened mesenchymal and/or possibly epithelial cells. There was moderate anisocytosis, the cytoplasm was granulated and eosinophilic. Epithelial cells, characterized by a finely vacuolated cytoplasm, were frequently observed. A moderate anisokaryosis was seen throughout the cells with mainly round nuclei accompanied by few large oval nuclei. Polynucleosis and anisonucleosis were also frequently observed. Special stains revealed multiple PAS positive accumulations within the epithelial cells. Immunohistochemistry was performed with monoclonal mouse anti-pan-cytokeratin antibody (Clone AE1/AE3) (1:50; Dako Agilent Pathology Solution, Glostrup, Denmark) coupled with polyclonal rabbit anti-mouse IgG antibody horseradish peroxidase (1:200; Dako Agilent Pathology Solution, Glostrup, Denmark) and 3,3′-diaminobenzidine (DAB) (Vector Laboratories, Burlingame, CA); with polyclonal rabbit anti-glial fibrillary acidic protein (anti-GFAP) antibody (1:500; Dako Agilent Pathology Solution, Glostrup, Denmark) and polyclonal rabbit anti-S100 antibody (1:400; Dako Agilent Pathology Solution, Glostrup, Denmark) coupled with biotinylated goat anti-rabbit IgG antibody (1:200; Vector Laboratories, Burlingame, CA); with avidin-biotin complex detection kit (Vectastain detection kit, Vector Laboratories, Burlingame, CA) and DAB (Vector Laboratories, Burlingame, CA) as chromogen.

Immunohistochemistry for cytokeratin was positive throughout the epithelial cells, while GFAP and S100 did not produce significant immunopositivity. Giving its similarities with the respiratory epithelium, a spinal neurenteric cyst (NC) was diagnosed.

Postoperative (PO) analgesia consisted of methadone (0.2 mg/kg BW/4 h IV) for the first day and buprenorphine (0.01 mg/kg BW/6 h IV) for the following 2 days. In addition, antibiotic medication (cephalexin 30 mg/kg BW/12 h PO) was administered for 5 days. The dog was discharged 4 days after surgery with the following medication: tramadol (2 mg/kg, BW/8 h PO), omeprazole (0.75 mg/kg BW/24 h PO), and firocoxib (5 mg/kg BW/24 h PO) for 5 days. Postoperatively, the dog was mildly ataxic in the hind limbs and showed a worsening of the monoparesis in the right hind limb, which improved over the following 2 months.

**Figure 4.**
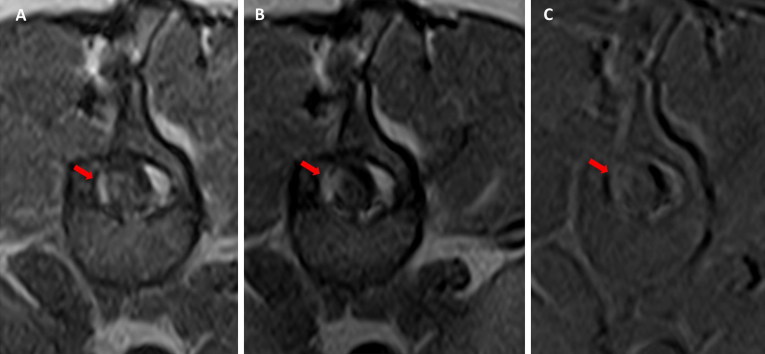
Case 1: Transverse T1-W pre (A) and post-contrast (B) images and subtraction (C) at the level of the surgical site. Note the asymmetrical meningeal thickening and enhancement (red arrow).

Seven months postoperatively, on neurological examination, the dog showed only a mild right hind limb monoparesis. MRI was performed at that time and revealed right dorsolateral localized meningeal contrast enhancement at the level of T13-L1 and an adjacent focal flattening of the spinal cord. A surgical scar tissue was suspected (Figure 4). The dog slowly continued to improve and, 3 years after surgery, the owner and the referring veterinarian reported that the clinical signs had stabilized and that a slight right hind limb monoparesis remained.

## Case 2

A 1-year-old entire male 27.8 kg mix-breed dog was presented with a history of acute onset of ataxia and paresis of the left hind limb, worsening over the following 10 days. At the first neurological examination, the dog was paraplegic, postural reactions were absent in the hind limbs, and segmental spinal reflexes were normal. Nociception was absent in the left hind limb and markedly reduced in the right hind limb. Neuroanatomical localization was T3-L3 myelopathy. The main differential diagnoses included inflammatory/infective (meningomyelitis of unknown origin; bacterial, fungal, viral, and parasitic myelitis), vascular (ischemic and hemorrhagic myelopathy) and, less likely, intervertebral disc extrusion or neoplastic (for example lymphoma and nephroblastoma) diseases.

MRI of the thoracolumbar vertebral column was performed using a 0.3 T Magnet (AIRIS II, Hitachi Medical System, Milan, Italy). Sequences acquired included T1-W and T2-W images in transverse and sagittal planes, T1-W images in transverse planes after intravenous administration of paramagnetic contrast media gadodiamide (Omniscan, 0.5 mmol/ml, GE Healthcare, Oslo, Norway) at 0.1 mmol/kg BW, fluid attenuated inversion recovery (FLAIR) in sagittal planes and short tau inversion recovery (STIR) in dorsal planes.

MRI showed an apparently intradural-intramedullary spinal cord abnormality at the level of the first lumbar vertebra which was hyperintense on T2-W and STIR images, hypointense on T1-W (Figure 1) and FLAIR and appeared, on T1-W post contrast images, to be surrounded by a hyperintense rim. In addition, cranially to the lesion and to the 7th thoracic vertebra, a dilatation of the central canal consistent with mild syringohydromyelia was detected.

Based on MRI findings, the main differential diagnoses included inflammatory disease such as an abscess, congenital anomaly (epidermoid cyst) or juvenile cystic neoplasia. At this time, the owner declined surgical intervention and the dog was treated with prednisolone (1 mg/kg BW/24 h PO). Three days after the first presentation, a subsequent examination showed worsening of the neurological signs with loss of nociception in both hind limbs and a surgical exploration of the lesion was planned. The dog was premedicated with fentanyl (0.002 mg/kg BW IV) and induced with propofol (5.5 mg/kg BW IV). General anesthesia was maintained with a total intravenous anesthesia by target-controlled infusion of propofol (20 mg/kg BW/h). During the surgery, analgesia was provided by a constant rate infusion of fentanyl (0.01 mg/kg BW/h IV).

A left-sided hemilaminectomy was performed. The exposed dura appeared swollen. A durotomy was performed revealing a reddish, well encapsulated mass attached to the spinal cord.

The mass was adherent to the spinal cord and, after pia mater resection, was isolated with the aid of microbipolar coagulation and dissected with microsurgical instruments and a surgical microscope (Zeiss nc31, Oberkochen, Germany). All the visible mass was removed, fixed in formalin, and submitted for histopathological examination. The specimen was processed and stained as the case 1.

Histology showed an encapsulated, cystic, and multiloculate tissue with moderately cellular epithelial proliferation embedded in moderate amount of stroma and surrounded by adipocytes. Cells were organized in a stratified cuboidal epithelium with cavities filled by mucoid material. In the same areas, there were ducts and tubules characterized by a ciliated respiratory epithelium. Distributed within the stroma were multiple microhemorrhages. Immunohistochemistry was negative for GFAP and positive for cytokeratin and epithelial cells showed PAS positive intracellular material (Figure 3). Post-operatively analgesia consisted of methadone (0.2 mg/kg BW/4 h IV) for the first day, methadone (0.2 mg/kg BW/6 h IV) for the following 2 days. The patient was treated with antibiotics (Pradofloxacin 5 mg/kg BW, once every 24 h PO) until discharge, 7 days later.

The dog regained nociception in the hind limbs within 2 days after the surgery. One week postoperatively, the patient was presented with an increased tone in the left hind limb, was able to ambulate unassisted showing proprioceptive ataxia and paraparesis with knuckling on the dorsum of the paw in the left hind limb. Two months after surgery, the hind limbs ataxia was less severe, and the dog did not show any more knuckling of the paw in the left hind limb.

Follow-up was obtained by telephone calls to the owner, who reported a slowly progressive continuous improvement. Two years after surgery, the owner sent a video, which showed the dog to be well ambulatory with very mild proprioceptive ataxia in the hind limbs.

## Discussion

In human medicine, NCs are rare congenital lesions of the central nervous system commonly found in the spinal cord or, more rarely intracranially in the caudal fossa (Gauden et al. 2012; Chakraborty et al. 2016). NCs result from the incomplete separation of the embryonic notochordal plate and endoderm during the third week of human development (Savage et al. 2010). The histopathological features resemble those of the epithelium of the gastrointestinal or respiratory tract and, for this reason, are described as enteric, endodermal, gastrogenic or bronchogenic cysts (Can et al. 2015).

NC in dogs is an extremely rare finding. To date only two intracranial (in the fourth ventricle) and two spinal NCs have been described (Molin et al. 2014; Ferrand et al. 2015; Alder et al. 2017; Kent et al. 2017).

In human medicine, the clinical signs may have a variable onset. An acute onset is thought to be related to a rapid increase in mucin secretion causing sudden spinal cord compression or due to haemorrhage within the cyst (Hicdonmez and Steinbok 2004). This scenario was likely in the second case described which showed an acute onset of clinical signs and microhaemorrhages within the cyst on pathology and to the authors knowledge this has not been previously described in a dog. The late onset and the progressive worsening over time are likely due to the slow growth rate of the ectopic tissue, as described in our first case and in the other published case studies.

In people, NCs can occur at any level of the spinal cord and are most commonly found in the lower cervical and upper thoracic spinal cord segments (Can et al. 2015). In humans, NCs have an intradural-extramedullary location in 95% of cases, often situated close to the ventral spinal cord. In less than 5% of all reported cases, NCs have an intramedullary localization (Jhawar et al. 2012). The two previous reports in veterinary medicine localized the NCs as extradural in one case and intradural-extramedullary in the other (Ferrand et al. 2015; Alder et al. 2017). In our patients, the cyst was intradural-extramedullary in case 1 and intradural-intramedullary in case 2 (Supplementary Video). An intradural-intramedullary location of an NC has not been previously described.

The MRI features of the NCs here reported, were similar to the previous descriptions consisting mainly in iso-hypointensity on T1-W and hyperintensity on T2-W images (Ferrand et al. 2015; Alder et al. 2017). Our cases, as described in human and previous veterinary cases, showed a mild peripheral contrast enhancement. This is hypothesized to be the result of a chronic inflammatory reaction due to leakage of liquid cyst content after spontaneous periodic tearing of the wall membrane (Molin et al. 2014; Yang et al. 2015).

MRI is the modality of choice for evaluating the lesions of the spinal cord (Dennis 2011). In human medicine, MRI has an excellent sensitivity and a moderate specificity for the diagnosis of intradural spinal lesion (Masciarelli et al 2017). In this cases, low field MRI provided adequate information regarding morphology of the lesion and allowed detection of associated cord changes, such as syringomyelia, but did not permit a precise localization or a definitive diagnosis as to the nature of the lesion. To date, the final diagnosis of NC can only be established after histopathological examination.

In human medicine, the epithelium of NC stains positive for epithelial membrane antigen, cytokeratin, and carcinoembryonic antigen, and negative for characteristic neuroectodermal markers such as GFAP (Gauden et al. 2012). These staining properties and the histopathological findings of respiratory/gastrointestinal epithelia strongly suggest that NCs are endodermal in origin (Zarineh et al. 2009). In our cases, the diagnosis of NC was supported by the positivity for PAS and diffuses cytokeratin immunoreactivity. Based on histopathologic features, human NCs are categorized into three types: type A cysts display a layer of cuboidal or columnar epithelium with or without cilia, on a basement membrane composed of type IV collagen; type B cysts show the same characteristics of type A, but have glands and may include mesenchymal tissue such as muscle, fat, lymphoid tissues, cartilage, and bone; type C cysts display the features of type A and include glial elements (Wilkins and Odom 1976). According to this classification, NCs of this report could be classified as type B cysts.

In humans, the treatment of choice for spinal NCs is complete surgical excision (Garg et al. 2008). Since complete resection is not always possible, other modalities of treatment can be performed including simple aspiration, fenestration, and/or marsupialization of the cyst. However, simple aspiration could lead to recurrence, while subtotal resection could induce adhesions with consequently postoperative neurological deterioration (Menezes and Traynelis, 2006).

In people, the recurrence rate of intramedullary NC after surgery was found to be 23%, which is probably an underestimation due to the lack of long-term follow-up in some reports (Kumar and Nayak 2002). The proliferation of non-resected epithelium as well as accumulation of cerebrospinal fluid (CSF) in the residual cavity of the cyst might lead the cyst to recur (Yang et al. 2015). This latter condition is considered the result of the development of iatrogenic pseudomeningocele, a rare complication of spinal surgery in humans (Macki et al. 2014).

The result of surgical treatment was excellent in both the reported cases. The second dog recovered ambulation despite loss of nociception for more than 48 h before surgery.

The use of the surgical microscope and microbipolar coagulation used in the second dog allowed better field visualization and cyst resection. In human spinal cord surgery, the use of surgical microscope, microsurgical techniques, and rigid attention to hemostasis are standard care for the surgical treatment (Gandhi and German 2013).

Spinal NC should be included in the list of differential diagnoses in young adult dogs with spinal cord dysfunction both with insidious and acute onset. Surgical treatment of spinal NC may lead to excellent results when complete resection is possible. No recurrence of clinical signs was seen in these cases in the period of follow up (greater than 2 years). MRI findings cannot distinguish spinal NCs from other pathologies, including neoplasms, which have a poorer prognosis. For this reason, surgical exploration should be considered in spinal patients with evidence of a suspected intradural lesion.
